# Cost of stroke: a controlled national study evaluating societal effects on patients and their partners

**DOI:** 10.1186/s12913-015-1100-0

**Published:** 2015-10-13

**Authors:** Poul Jennum, Helle K. Iversen, Rikke Ibsen, Jakob Kjellberg

**Affiliations:** Danish Center for Sleep Medicine, Department of Clinical Neurophysiology, Faculty of Health Sciences, University of Copenhagen, Glostrup Hospital, DK 2600, Copenhagen, Glostrup Denmark; Stroke Unit, Department of Neurology, Faculty of Health Sciences, University of Copenhagen, Glostrup Hospital, Copenhagen, Denmark; 2minds, Klosterport 4E, 4, Aarhus, Denmark; Danish Institute for Health Services Research, Copenhagen, Denmark

**Keywords:** Stroke, Illness and health care costs, Economic burden, Employment, Partner, Social

## Abstract

**Background:**

To estimate the direct and indirect costs of stroke in patients and their partners.

**Description:**

Direct and indirect costs were calculated using records from the Danish National Patient Registry from 93,047 ischemic, 26,012 hemorrhagic and 128,824 unspecified stroke patients and compared with 364,433, 103,741 and 500,490 matched controls, respectively.

**Results:**

Independent of age and gender, stroke patients had significantly higher rates of mortality, health-related contacts, medication use and lower employment, lower income and higher social-transfer payments than controls. The attributable cost of direct net health care costs after the stroke (general practitioner services, hospital services, and medication) and indirect costs (loss of labor market income) were €10,720, €8,205 and €7,377 for patients, and €989, €1,544 and €1.645 for their partners, over and above that of controls for hemorrhagic, ischemic and unspecified stroke, respectively. The negative social- and health-related status could be identified up to eleven years before the first diagnosis.

**Conclusion:**

Stroke has significant mortality, morbidity and socioeconomic consequences for patients, their partners and society.

## Background

A significant proportion of the population will experience stroke in their lifetime. The outcomes of stroke range from full remission to severe disability and death, and the event causes significant personal, familiar and societal burdens.

In the past 20 years significant progress has been made towards identifying risk factors and the early stages of stroke, managing the condition, and developing early primary and secondary interventions. Stroke is associated with high cardiovascular risk factors, including hypertension, smoking, sedentary lifestyle, obesity, reduced quality of life, depression or anxious mood, low income, social interaction, and social status [1]. Previous studies examining the burden of stroke have focused on the direct costs, e.g., hospital services and the use of treatment procedures, such as medication, thrombolysis, physiotherapy, etc., as well as the reduction in quality of life, and indirect costs [2–7]. Most studies have evaluated case series, models, or quality of life. A number of studies have sought to evaluate the impact of stroke at a national level [8–13], and upon caregivers after the stroke [14–17]. However, no studies have evaluated the health and social burden before the stroke on patients and their partners in terms of the total socioeconomic costs before and after the stroke incident.

The objective of the current study was to use a national patient database to evaluate the cost of stroke to patients, their partners and society, and, using a case–control design, to examine the associations of pre-existing morbidity, social factors, and economic status with stroke and its consequences.

## Construction and content

The NPR contains information about diagnosis, procedures (diagnostic, treatment). The National Health Security contains all information regarding healt contacts in primary sector and the Danish Medicine Agency includes all information regarding prescripting medication.

## Utility and Discussion

All data are recorded in the dataset by each health contact in the healthcare sector for all national patients. The user interface is recorded via a dedicated software systems.

## Methods

In Denmark, it is possible to calculate health sector costs and productivity losses related to diseases because information from public and private hospitals, general practice, and privately practicing specialists, and data about medication, social transfers, labor market income and employment for all Danes are registered in central databases. Health care in Denmark is predominantly publicly funded, and all patient contacts with the hospital system are recorded in the Danish National Patient Registry (NPR) at the time of contact; this information includes the primary diagnosis [18]. The NPR is a time-based national database that includes data from all inpatient and outpatient contacts, which means that the data we can extract are representative of all patients in Denmark who have received a diagnosis of stroke in the primary or secondary sector in a public or private hospital. Since data are available for the entire observation period, we can trace patients retrospectively and prospectively, relative to the time of the diagnosis. Furthermore, all contacts in the primary sector (general practice and specialist practices) and the use of medications are recorded in the databases of the National Health Security and the Danish Medicine Agency, respectively. All national patients with a diagnosis of stroke are included.

The economic consequences of stroke for patients and their partners were estimated by determining the yearly cost of illness per patient diagnosed with the ICD-10 code for stroke (I61: cerebral hemorrhage; I63: brain infarction; I64: stroke not otherwise defined whether due to infarction or hemorrhage); these codes are assigned after patient evaluation in each hospital (based on a standardized evaluation of stroke), and comparing the figure with that of a matched control group. The model for deriving these estimates has been described elsewhere [19–22].

By reviewing the NPR, we identified all patients who received a first diagnosis of stroke between 1997 and 2009. Then, using data from the Civil Registration System Statistics Denmark database (which includes information about social factors, marital and cohabiting status, incomes, pensions, etc.) [23], we randomly selected citizens of the same age and sex as the patients. Social compensation was taken into account by selecting control subjects who resided in the same area of the country in which the patients lived, and who had the same civil status. The ratio of control subjects to patients was 4:1. Data from patients and matched control subjects who could not be identified in Statistics Denmark using the Civil Personal Registration (CPR) System database were excluded from the sample. More than 99 % of the observations in the two groups were successfully matched. Patients and matched control subjects were followed from the year of diagnosis until 2009. Thus, patients with a stroke at the beginning of the period contributed follow-up data over 11 years; those experiencing a stroke at the end of the period provided pre-data, and all those in between provided varying amounts of both pre- and post-data. If a patient or control was not present in the CPR register on January 1^st^ each year, then the corresponding control or patient control was not included in the dataset for that year. Patients absent from the CPR register are typically deceased, in prison, or have emigrated to another country. All patients’ partners (married or unmarried, and irrespective of gender) were also identified. A similar control group of partners was identified on the basis of age, gender and socio-demographic status.

The health care costs were then divided into annual direct and indirect health care costs. Direct costs, including hospitalization and outpatient costs, were calculated using diagnosis-related group (DRG) weights, and specific outpatient tariffs. These cost estimates were all based on data from the Danish Ministry of Health. The use and costs of drugs were derived from data from the Danish Medicines Agency. The retail price of each drug (including dispensing costs) was multiplied by the number of transactions. The frequencies and costs of consultations with general practitioners and other specialists were based on data from the National Health Security.

The indirect costs, which are those related to reduced labor supply, are based on figures from Danish Income Statistics. Social-transfer payments, which in Denmark are primarily publicly funded, were also included; they include subsistence allowances, pensions, social security, social assistance, publicly funded personal support for education, and others. Cost-of-illness studies measure the economic burden resulting from disease and illness across a defined population and include direct and indirect costs. Direct costs are the value of resources used for the treatment, care and rehabilitation of people with the condition under study. Indirect costs are the value of economic resources lost through disease-related work disability or premature mortality. As patients leave the national data registers at the time of death, the indirect cost estimate comprises only the production loss related to disease-related work disability. The human capital approach is used to measure the productivity costs based on the difference in labor market income between the cases and the matched controls. It is important to distinguish costs from monetary transfer payments such as disability and welfare payments. These payments represent a transfer of purchasing power to the recipients from general taxpayers but do not constitute net increases in the use of resources and are, therefore, not included in the total cost estimate.

In the Danish Stroke Register (formerly the National Indicator Project, NIP), it was possible to choose the diagnoses of ischemic stroke, hemorrhagic stroke or unspecified stroke. Although all patients with stroke underwent a CT or MRI scan, some stroke units customarily classified ischemic strokes as unspecified strokes, and these were the diagnoses reported to the Danish National Patient Registry. In the present study, a total of 247,883 strokes were considered. The expected distribution of stroke types would be 15 % hemorrhagic strokes, corresponding to 37,183 patients. In fact, only 26,012 patients (10.5 % of the total) were registered as hemorrhagic strokes, so 11,171 patients with hemorrhagic stroke were assigned to the unspecified group, equivalent to 9 % of the unspecified group, although 91 % were ischemic strokes. The register no longer permits a diagnosis of unspecified stroke, and in 2014 the distribution was 87 % ischemic and 13 % hemorrhagic strokes. The diagnoses have been validated previously [24].

Costs were measured on a yearly basis and adjusted to 2009 prices using the general price index. All costs were measured in DKK and converted to Euros (€1 = DKK 7.45).

Survival was analyzed by the Kaplan-Meier method. The resulting survival curves take into account the censoring of data that arises since not all patients died before the end of the analysis period. The survival curves are stratified into case and control groups, and show the estimates of the survival distribution function.

The study was approved by the Danish Data Protection Agency. Data were handled in a manner that did not reveal the identity of any patients or control subjects, so neither individual nor ethical approval was required. Statistical analysis was done with SAS 9.1.3 (SAS, Inc., Cary, NC). Statistical significance of the cost estimates was assessed by nonparametric bootstrap analysis (33).

## Results

93,047 ischemic, 26,012 hemorrhagic and 128,824 stroke NOS patients were registered. They were compared with 364,433, 103,741 and 500,490 matched controls, respectively, identified from the National Danish Patient Registry. In addition, partners of patients in the case group were matched with partners in the matched control group. Almost half of the patients in the patient group had a partner. Mean (95 % CI) ages were 68.5 (68.3–68.7), 79.9 (79.8–71.0), and 72.2 (72.1–7.3) years for ischemic, hemorrhagic, and NOS stroke, respectively (Table 1).

**Table 1:** Age distribution of stroke patients included in the study

	Hemorrhagic stroke			Ischemic stroke			Stroke (NOS)		
Age	Patients	Controls	%	Patients	Controls	%	Patients	Controls	%
<20	296	1,184	1.1	285	1,139	0.3	102	408	0.1
20–29	344	1,375	1.3	376	1,504	0.4	571	2,282	0.5
30–39	662	2,647	2.6	1,300	5,194	1.4	1,877	7,503	1.5
40–49	1,766	7,063	6.8	4,621	18,450	5.1	5,775	23,041	4.6
50–59	3,587	14,328	13.8	11,847	47,140	12.9	14,494	57,578	11.5
60–69	5,188	20,715	20.0	20,028	78,998	21,7	24,817	97,675	19.5
70–79	6,940	27,670	26.7	26,674	104,277	28.6	37,310	144,910	29.0
≥80	7,229	28,759	27.7	27,916	107,731	29.6	43,878	167,093	33.4
All	26012	103,741	100	93,047	364,433	100	128,824	500,490	100

*NOS* not otherwise specified

### Survival in stroke

**Fig. 1:**
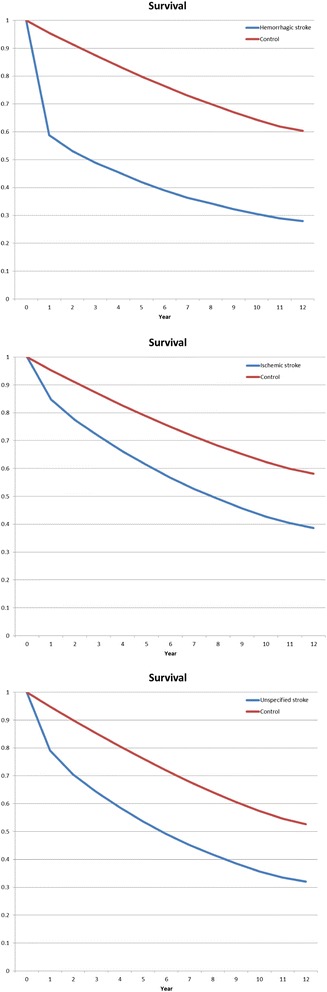
Survival after a diagnosis of hemorrhagic stroke (top), ischemic stroke (middle) and stroke not otherwise specified (bottom) compared with controls

Ten-year survival was significantly lower in stroke patients than in their respective controls (*p* < 0.001): 0.305 (95 % CI, 0.298–0.312) *vs.* 0.643 (0.539–0.647) for hemorrhagic, 0.428 (0.423–0.432) *vs.* 0.624 (0.621–9.626) for ischemic, and 0.357 (0.354–0.360) *vs.* 0.574 (0.572–0.576) for unspecified stroke patients (all comparisons, *p* < 0.0001). Twelve-year survival for hemorrhagic, ischemic stroke and stroke NOS is shown in Fig. 1. Lower survival tended to reduce the total health care costs. We did not adjust for the potential costs of those people who died since there is no accepted model for evaluating diseases with high mortality.

### Direct costs from outpatient, inpatient and primary care, and drug consumption

More patients and their partners than respective control subjects were treated in outpatient clinics, were hospitalized, used medication and had contact with the primary care system (Tables 2 and 3).

**Table 2:** Distribution of health care consumed and income in stroke patients after diagnosis and in corresponding controls (percentage)

	Hemorrhagic stroke			Ischemic stroke			Stroke NOS		
	Case	Control	*P*	Case	Control	*P*	Case	Control	*P*
Outpatient treatment	59.2	36.6	<0.001	59.4	37.5	<0.001	59.3	38.1	<0.001
Inpatient treatment	57.9	20.5	<0.001	54.8	21.6	<0.001	54.2	22.5	<0.001
Medication	93.3	85.1	<0.001	97.8	86.3	<0.001	97.3	87.4	<0.001
Public health insurance	97.4	95.7	<0.001	99.0	96.0	<0.001	98.8	96.0	<0.001
Income from employment	21.0	28.6	<0.001	18.2	24.5	<0.001	16.2	21.4	<0.001
Public transfer income total	86.6	77.9	<0.001	89.8	81.7	<0.001	90.3	84.0	<0.001
*Pension*	*55.9*	*60.0*	<0.001	*64.4*	*65.8*	<0.001	*67.7*	*70.3*	<0.001
*Other public transfers*	*25.3*	*15.3*	<0.001	*21.2*	*14.2*	<0.001	*19.3*	*12.2*	<0.001
*Sickpay (public-funded)*	*9.8*	*4.2*	<0.001	*7.8*	*3.3*	<0.001	*6.1*	*2.8*	<0.001

*NOS* not otherwise specified

**Table 3:** Health care costs of stroke in patients before and after diagnosis

	Hemorrhagic	Control	*P*	Ischemic	Control	*P*	Stroke NOS	Control	*P*
Before diagnosis									
* N*	141,389	562,781		547,465	2,129,024		628,432	2,416,189	
Outpatient treatment	341	233	<0.001	315	240	<0.001	335	234	<0.001
Inpatient treatment	1,758	1,030	<0.001	1,539	1,019	<0.001	1,715	1,033	<0.001
Medication	592	466	<0.001	600	475	<0.001	649	478	<0.001
Public health insurance	335	283	<0.001	325	284	<0.001	353	288	<0.001
Income from employment	9.654	12.469	<0.001	9,438	11,908	<0.001	8,413	11,213	<0.001
Public transfer income total	11,991	10,877		12,333	11,365		12,804	11,645	
* Pension*	*7,936*	*7,814*	<0.001	*8,360*	*8,187*	<0.001	*8,979*	*8,722*	<0.001
* Other public transfers*	*3,728*	*2,850*	<0.001	*3,678*	*2,979*	<0.001	*3,533*	*2,736*	<0.001
* Sickpay (public-funded)*	*328*	*213*	<0.001	*295*	*199*	<0.001	*292*	*187*	<0.001
Direct health care costs	3,026	2,013		2,778	2,018		3,051	2,032	
Indirect costs, foregone earnings	2,815			2,470			2,800		
Sum of direct and indirect costs	5,840	2,013		5,248	2,018		5,851	2,032	
Net costs	3,827			3,231			3,819		
Social transfer payments	11,991	10,877		12,333	11,365		12,804	11,645	
After diagnosis									
* N*	89,043	569,616		398,140	1,869,080		566,620	2,862,856	
Outpatient treatment	664	396	<0.001	679	424	<0.001	665	409	<0.001
Inpatient treatment	7,840	1,814	<0,001	5,986	1,906	<0.001	5,904	1,981	<0.001
Medication	924	605	<0.001	1,134	632	<0.001	1,119	642	<0.001
Public health insurance	659	354	<0.001	632	363	<0.001	609	363	<0.001
Income from employment	6,165	9,965	<0.001	5,196	8,294	<0.001	4,875	7,350	<0.001
Public transfer income total	13,395	12,051		14,149	12,832		14,070	13,185	
* Pension*	8,328	9,693	<0.001	10,091	10,650	<0.001	10,306	11,225	<0.001
* Other public transfers*	4,222	2,149	<0.001	3,462	2,015	<0.001	3,289	1,816	<0.001
* Sickpay (public-funded)*	846	209	<0.001	596	167	<0.001	474	145	<0.001
Direct health care costs	10,088	3,168		8,431	3,324		8,297	3,395	
Indirect costs, foregone earnings	3,800			3,098			2,475		
Sum of direct and indirect costs	13,888	3,168		11,529	3,324		10,772	3,395	
Attributable costs	10,720			8,205			7,377		
Social transfer payments	13,395	12,051		14,149	12,832		14,070	13,185	

*NOS* not otherwise specified

### Indirect costs, social costs, employment rate and income

**Fig. 2:**
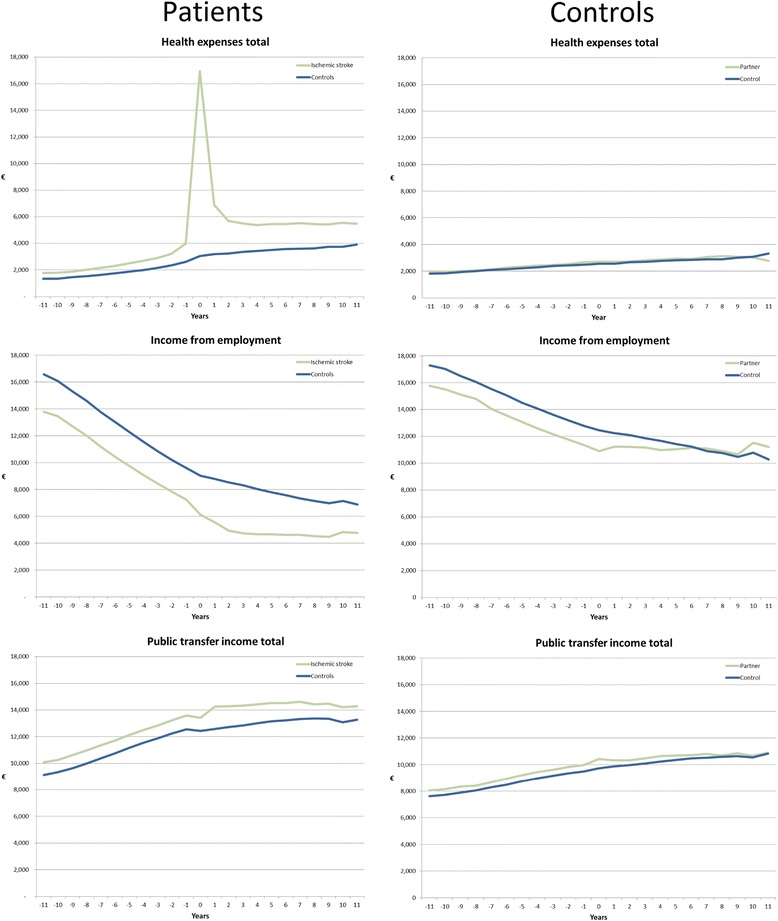
Total health expenses, income from employment, and social transfer of income in patients with ischemic stroke and their partners compared with respective controls before and after diagnosis (patients with hemorrhagic and stroke NOS show similar patterns)

Greater proportions of patients and their partners received help from social services compared with control subjects and their partners. The statistically significant differences in employment rates (Tables 4 and Fig. 2) and income from employment were smaller before the injury, whereas the highest social-transfer rates were seen before the diagnosis and increased thereafter (not shown). Income increased in partners after the patients’ injury relative to controls (Fig. 2).

**Table 4:** Distribution of health care received and income in partners after diagnosis and in corresponding controls (percentage)

	Hemorrhagic stroke			Ischemic stroke			Stroke NOS		
	Partner	Control	*P*	Partner	Control	*P*	Partner	Control	*P*
Outpatient treatment	34.3	35.2	<0.001	36.4	35.6	0.067	59.3	38.1	<0.001
Inpatient treatment	16.2	16.4	0.169	17.3	16.7	<0.001	54.2	22.5	<0.001
Medication	83.8	84.3	0.001	86.7	85.6	<0.001	97.3	87.4	<0.001
Public health insurance	96.8	96.8	0.100	97.1	97.0	0.656	98.8	96.0	<0.001
Income from employment	40.5	37.8	<0.001	33.1	34.3	<0.001	16.2	21.4	<0.001
Public transfer income total	70.9	71.0	0.012	76.9	74.0	<0.001	90.3	84.0	<0.001
*Pension*	*43.5*	*47.6*	<0.001	*50.4*	*51.1*	<0.001	*67.7*	*70.3*	<0.001
* Other public transfers*	*22.7*	*20.0*	<0.001	*23.3*	*20.3*	<0.001	*19.3*	*12.2*	<0.001
* Sickpay (public-funded)*	*7.9*	*6.1*	<0.001	*6.0*	*5.1*	<0.001	*6.1*	*2.8*	<0.001

*NOS* not otherwise specified

### Total health care costs per year

The sources of information and the average annual health care cost per person-year by cost category for patients with stroke in Denmark compared with age- and sex-matched control subjects before and after diagnosis are presented in Table 4; the corresponding numbers for their partners are shown in Table 5 (supplementary file). The attributable cost of direct net health care costs after the stroke (general practitioner services, hospital services, and medication) and indirect costs (loss of labor market income) were €10,720, €8,205, and €7,377 for patients, and €989, €1,544, and €1.645 for their partners (Table 5) over and above that of controls for hemorrhagic, ischemic, and unspecified stroke, respectively. The majority of the difference in health care costs for the patients were incurred in the first year of the disease, when the differences in health care costs were €17.220, €13.913, and €13.259. The average incremental health care costs during the first 5 years after the stroke were €2.771, €2.515, and €2.432.

**Table 5:** Health care costs and income of partners of patients before and after diagnosis (Euros)

	Hemorrhagic	Control	*P*	Ischemic	Control	*P*	Stroke NOS	Control	*P*
Before diagnosis									
* N*	80,184	327,758		315,730	1,256,233		345,920	1,366,131	
Outpatient treatment	272	265	0.163	276	271	0.087	284	272	<0.001
Inpatient treatment	1,267	1,218	0.100	1,317	1,237	<0.001	1,370	1,283	<0.001
Medication	459	449	0.002	479	459	<0.001	487	465	<0.001
Public health insurance	294	287	<0.001	292	289	<0.001	301	295	<0.001
Income from employment	14,029	14,946	<0.001	12,938	14,374	<0.001	12,471	14,000	<0.001
Public transfer income total	8,955	8,686		9,257	8,808		9,409	8,956	
* Pension*	*5,336*	*5,131*	<0.001	*5,271*	*5,172*	<0.001	*5,666*	*5,492*	<0.001
* Other public transfers*	*3,301*	*3,250*	0.054	*3,674*	*3,353*	<0.001	*3,458*	*3,197*	<0.001
* Sickpay (public-funded)*	*318*	*304*	0.410	*312*	*282*	<0.001	*285*	*267*	<0.001
Direct health care costs	2,291	2,219		2,364	2,256		2,441	2,315	
Indirect costs, foregone earnings	917			1,436			1,529		
Sum of direct and indirect costs	3,208	2,219		3,800	2,256		3,970	2,315	
Attributable costs	989			1,544			1,654		
Social transfer payments	8,955	8,686		9,257	8,808		9,409	8,956	
After diagnosis									
* N*	43,817	300,413		203,082	978,759		274,066	1,403,525	
Outpatient treatment	366	394	0.010	410	409	1,000	399	399	1,000
Inpatient treatment	1,373	1,380	1,000	1,471	1,408	<0.001	1,548	1,472	<0.001
Medication	488	509	<0.001	555	531	<0.001	565	548	<0.001
Public health insurance	345	344	1,000	360	356	<0.001	362	360	0.010
Income from employment	14,429	13,522	<0.001	11,082	11,841	<0.001	10.908	11,146	<0.001
Public transfer income total	9,643	9,575		10.492	10.082		10.480	10.217	
* Pension*	6,169	6,623	<0.001	6,961	7,118	<0.001	7,086	7,406	<0.001
* Other public transfers*	3,067	2,644	<0.001	3,227	2,713	<0.001	3,108	2,584	<0.001
* Sickpay (public-funded)*	407	308	<0.001	304	251	<0.001	287	227	<0.001
Direct health care costs	2,571	2,627		2,796	2,704		2,874	2,778	
Indirect costs, foregone earnings	(907)			759			238		
Sum of direct and indirect costs	1,665	2,627		3,555	2,704		3,112	2,778	
Attributable costs	(962)			851			334		
Social transfer payments	9,643	9,575		10.492	10.082		10.480	10.217	

NOS: not otherwise specified

Social-transfer payments were all significantly greater in stroke patients than in control subjects.

### Influence of age and sex on employment and direct and indirect costs

The relationships between age and sex on one hand, and direct and indirect costs on the other were stronger after the diagnosis in patients than in controls, for all age groups. On average, male patients and controls had higher incomes than their female counterparts. Age and sex had pronounced effects on direct costs; in particular, there were greater expenses due to hospitalization and medication among younger patients, whereas direct and indirect costs were higher among younger adults, due to increased indirect costs (data for ischemic stroke are shown in Fig. 2 for patients and their controls; the patterns for the other stroke diagnoses were similar).

## Discussion

The study has several important findings. Stroke patients already present a significant burden before their stroke. Stroke has significant economic consequences: patients and their partners had significantly higher rates of contact with all sectors of the health care system than did age- and sex-matched control subjects. This covers contact with general practice, outpatient clinics, and in-hospital services, as well as medication use, and publicly supported payment for medication. Total expenses for stroke patients were more than four times those for the controls. Patients had lower employment rates but received welfare payments significantly more often than controls. Employed patients had lower incomes than employed control subjects. However, the cost estimates are lower, as some previous studies have found. This is probably because 1) we compared patients with a control group who may have diseases other than stroke, 2) the study did not include the cost of home nursing and nursing homes, and 3) in the aging population under consideration, a higher proportion of patients than controls are on a pension, which reduces the indirect costs.

Stroke has a significant influence on patients’ partners: health care contacts were more frequent in the secondary health care system, in which partners made greater demands on the social care system, and consequently incurred greater direct and indirect costs. Stroke causes significant mortality and morbidities; the mortality rates in the data are presented, but we were not able to include loss of life in the cost estimates as there are no valid methods for estimating these consequences.

It is of particular note that stroke patients and their partners tended to experience pre-existing social, economic and morbidity effects several years before the incident. These effects are considerable and present more than 10 years before the stroke incident. This is to be expected since strokes are often the consequence of major cardiovascular and other risk factors (e.g., lifestyle factors), so that stroke cases often suffer from other comorbidities.

The differences between patients with stroke and control subjects were as considerable as those for patients with chronic neurological diseases [19–22, 25]. Given the large numbers of people involved and the differential effects by age and gender, the personal, familiar and societal costs are considerable.

Previous studies have documented the effect of stroke on partners with respect to quality of life, perceived health, and social effects [7, 15, 17, 26–30]. We found that stroke had a significant impact on health consumption and social effects on caregivers, leading to higher direct and indirect costs. They arose from increased health care usage and lower income levels. Partners’ incomes did not increase to compensate for the drop in the income of patients with chronic disease, and, in fact, actually tended to decrease. Such effects have been observed for people with serious fatal neurological diseases like amyotrophic lateral sclerosis, whose partners compensate for the loss of social welfare by increasing their own income level. [31] This is probably due to several factors: the disease also affects younger individuals, causes loss of social competences, and there may be more focus on families than on stroke patients. However, stroke sequelae affect the partner in several ways, resulting in increased morbidity and use of medication, and work capability and income.

At the time of the diagnosis, patients already presented significant role limitations. Such limitations have been described in other chronic and progressive disorders, such as multiple sclerosis [32], Parkinsonism, narcolepsy [33], and sleep-disordered breathing, although these are associated with long-lasting and pre-diagnostic courses. This cannot be explained solely by the stroke. Stroke patients, reported to the NPR, showed significant worsening of social and health care costs variables a long time before their diagnosis. This difference cannot be explained by the choice of the control group since we selected the group from similar social areas. The pre-existing socioeconomic load may instead be associated with the occurrence of other comorbidities, social factors, and fatigue [34, 35], amongst other factors. Further evaluation of pre-existing comorbidities should enable these issues to be addressed.

The occurrence and management of stroke tend to follow a social gradient [36, 37], which we have further investigated in this study. In order to control potential negative social effects of stroke we used selection of controls using geographic but not full social compensation (e.g., by income or education).

### Limitations of the study

The current study included all patients who had received a diagnosis of stroke. A study of the registration procedure suggests that approximately 85–90 % of registrations in the NPR are correct [38]. However, it is likely that some diagnoses are erroneous due to incorrect registration, or variation in the registration procedure or diagnostic criteria [24].

Stroke patients are likely to have a high registration rate in the national register, but rates may be underestimated through the failure to recognize patients with early or minor symptoms, or those who have contact with the primary sector, since such contacts are registered without a diagnosis. In general, the sample in the database comprises almost the entire national patient population. As the study is based on registry data only resource use covered by these registers is included in the analysis. Danish registers do not cover home nursing or nursing homes, so the cost estimates do not quantify the full cost of the disease.

## Conclusion

Stroke causes significant mortality. Stroke survivors and their relatives experience significant consequences in terms of increased morbidity and, in turn, significantly higher health-related and social-transfer costs and slightly lower levels of employment and income compared with a matched control group of people without a diagnosis of stroke. The consequences for partners are not negligible and account for approximately one-third of the total familiar costs. Costs are highest among younger adults due to loss of work and the resulting higher indirect costs. Stroke patients have significantly more health-related contacts and present social vulnerability years before their incident. The significance of morbidities prior to the onset of stroke should be examined further to identify and treat groups at high risk of developing chronic disease so that its costs and consequences for patients, their families and society can be reduced.

## Abbreviations

NOS: Not otherwise specified
NPR: National Patient Registry
